# Phenyl Acyl Acids Attenuate the Unfolded Protein Response in Tunicamycin-Treated Neuroblastoma Cells

**DOI:** 10.1371/journal.pone.0071082

**Published:** 2013-08-15

**Authors:** Marta Zamarbide, Eva Martinez-Pinilla, Ana Ricobaraza, Tomás Aragón, Rafael Franco, Alberto Pérez-Mediavilla

**Affiliations:** 1 Cell and Molecular Neuropharmacology Laboratory, Neurosciences Division, Center for Applied Medical Research - CIMA, University of Navarra, Pamplona, Spain; 2 Department of Biochemistry and Genetic, University of Navarra, Pamplona, Spain; 3 Gene Therapy Division, Center for Applied Medical Research – CIMA, University of Navarra, Pamplona, Spain; 4 Department of Biochemistry and Molecular Biology, University of Barcelona, Barcelona, Spain; 5 Laboratoire de Neurobiologie, ESPCI-CNRS UMR 7637, ESPCI-ParisTech, Paris, France; Massachusetts General Hospital/Harvard Medical School, United States of America

## Abstract

Understanding how neural cells handle proteostasis stress in the endoplasmic reticulum (ER) is important to decipher the mechanisms that underlie the cell death associated with neurodegenerative diseases and to design appropriate therapeutic tools. Here we have compared the sensitivity of a human neuroblastoma cell line (SH-SY5H) to the ER stress caused by an inhibitor of protein glycosylation with that observed in human embryonic kidney (HEK-293T) cells. In response to stress, SH-SY5H cells increase the expression of mRNA encoding downstream effectors of ER stress sensors and transcription factors related to the unfolded protein response (the spliced X-box binding protein 1, CCAAT-enhancer-binding protein homologous protein, endoplasmic reticulum-localized DnaJ homologue 4 and asparagine synthetase). Tunicamycin-induced death of SH-SY5H cells was prevented by terminal aromatic substituted butyric or valeric acids, in association with a decrease in the mRNA expression of stress-related factors, and in the accumulation of the ATF4 protein. Interestingly, this decrease in ATF4 protein occurs without modifying the phosphorylation of the translation initiation factor eIF2α. Together, these results show that when short chain phenyl acyl acids alleviate ER stress in SH-SY5H cells their survival is enhanced.

## Introduction

The administration of 4-phenylbutyrate (PBA) is indicated as adjunct therapy in the chronic management of patients with disorders of the urea cycle that involve deficiencies of either carbamoylphosphate synthetase, ornithine transcarbamoylase or argininosuccinate synthetase [1], [2]. Phenylacetate produced by PBA metabolism may be conjugated to glutamine to form phenylacetylglutamine, which serves as an alternative to urea in ammonia excretion. Furthermore, PBA is potentially beneficial in the treatment of sickle cell disease, thalassemia, cancer, cystic fibrosis, spinal muscular atrophy, amyotrophic lateral sclerosis and type 2 diabetes mellitus [3]–[11]. In these pathologies, PBA appears to enhance both gene transcription and protein synthesis due to its properties as an histone deacetylase (HDAC) inhibitor [12].

Protein misfolding and aggregation are known to be associated with pathologies like Alzheimer's, Parkinson's or Huntington's diseases [13]. Interestingly, PBA produces beneficial effects in animal models of these neurodegenerative diseases, both by inhibiting HDACs and by acting as a chemical chaperone that reduces the stress in the endoplasmic reticulum (ER) caused by huntingtin, alpha-synuclein, p-tau or beta-amyloid (see [12] for review). It was recently shown that PBA protects against ER stress-induced neuronal cell death [14], an effect that was correlated with a marked increase in H3 histone acetylation and a decrease in the expression of glucose-regulated protein 94 (GRP94), whose transcription augments in response to defects in N-linked glycosylation [15].

**Figure 1:**
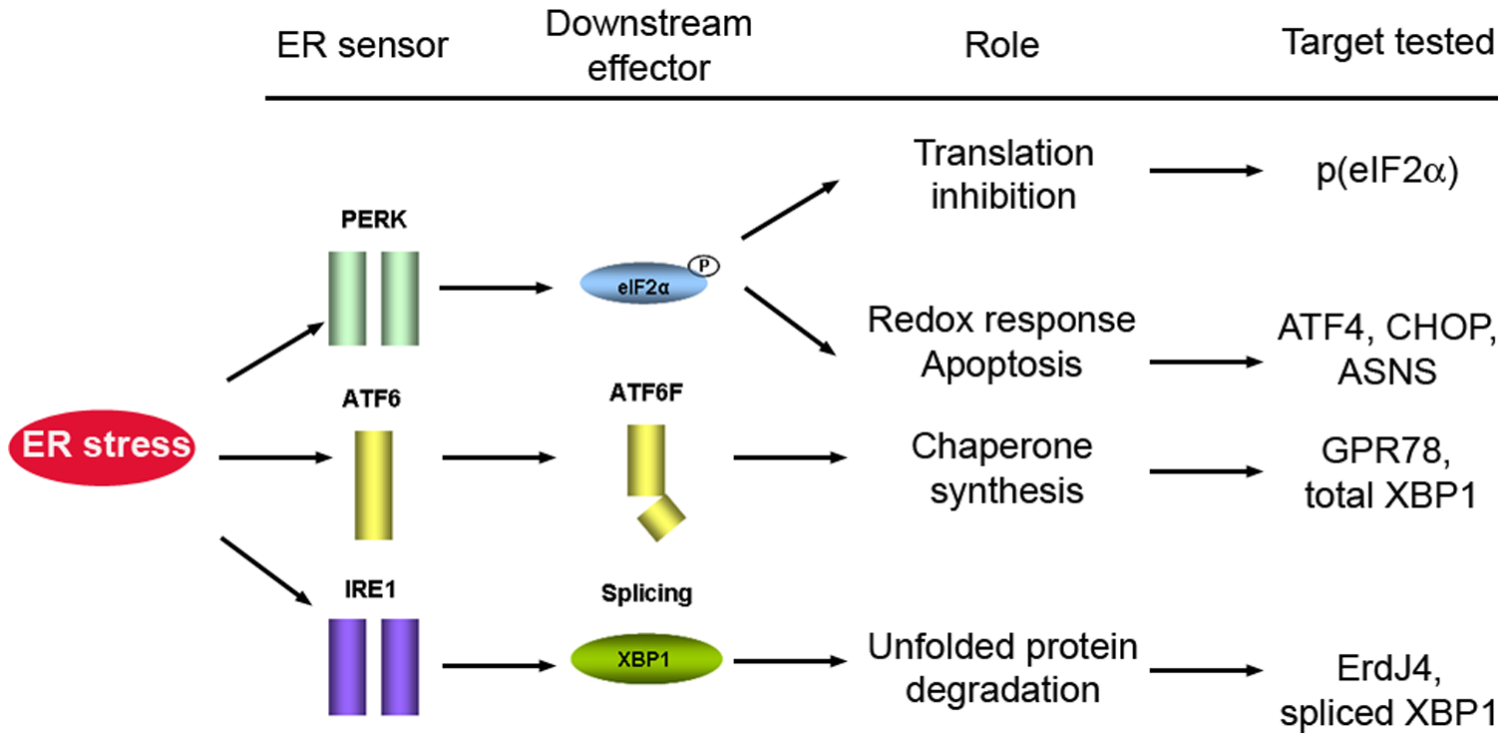
Scheme showing the three major ER-stress sensors: PERK, ATF6 and IRE1 and the link to the targets assayed in this report. Activated PERK phosphorylates eIF2α to attenuate protein translation but allowing the expression of ATF4-dependent genes, CHOP and ASNS, involved in redox- and apoptosis-related pathways. Cleaved -active- ATF6 leads to induction of molecular chaperones (GPR78) and of the transcription factor XBP1. IRE1 activation leads to XBP1 splicing, transcriptional activation of chaperones and stimulation of protein degradation through ErdJ4, which is a component of the ER-associated degradation (ERAD) system.

N-linked glycosylation is a key step early in the folding of most proteins that takes place within the endoplasmic reticulum. Tunicamycin is a drug that inhibits this process and that provokes the accumulation of misfolded proteins in the ER, the main hallmark of ER stress. In turn, ER stress triggers a compensatory mechanism, the unfolded protein response (UPR), and the three independent transmembrane ER stress sensors activated by ER stress – IRE1, PERK and ATF6– initiate three independent signaling mechanisms that converge on an integrated program of gene expression aimed to provide relief (Fig. 1). The most conserved step in UPR signaling is the non-conventional splicing of XBP1 mRNA that is initiated by IRE1, a transmembrane ER resident protein that contains a kinase and endonuclease domain in its cytosolic region. Upon activation, the Ire1 RNAse domain can excise a 26 nucleotide intron from within the mRNA coding for the XBP-1 transcription factor. Translation of this alternatively spliced XBP1 mRNA produces a very active transcription factor that drives the transcription of a vast set of UPR genes. IRE1/XBP1 splicing is the most highly conserved signaling component of the UPR and it is critical to determine the fate of cells in response to ER stress [16], [17]. However, the role of this splicing event in neurodegeneration still remains controversial.

Several other genes are known to be involved in the UPR. For example, following its synthesis activating transcription factor 6 (ATF6) is anchored to the surface of the endoplasmic reticulum through a C-terminal transmembrane domain. ER-localized ATF6 is inactive, although ER deficiencies drive the translocation of this protein to the Golgi apparatus, where it is processed by site1 and site2 proteases [18]. This processing releases the functional ATF6c transcription factor which then travels to the nucleus to activate the transcription of chaperone genes [19]. The multidomain transmembrane protein PERK also contains an ER stress sensor and a kinase domain in its cytosolic region. ER stress causes the clustering of PERK in the plane of the endoplasmic reticulum and the activation of its kinase domain, which phosphorylates the translation initiation factor eIF2α and dampens protein synthesis. PERK-dependent inhibition of cellular translation alleviates the protein load in the ER as the phosphorylation of eIF2α impairs the translation of most mRNAs. However, a small subset of messenger RNAs containing small upstream open reading frames (uORFs) benefit from eIF2α phosphorylation and they are preferentially translated under these conditions. The ATF4 transcription factor is one such protein and it activates a subset of transcripts that either enhance the folding capacity of the ER or promote apoptosis [20].

Here, we examine the activation of UPR signaling in response to tunicamycin in the SH-SY5H cell line, and the cell death caused by this insult. Based on this characterization, we examined the capacity of two phenyl acids, PBA and 5-phenylvalerate (PVA), to quash UPR signaling and improve the survival of these neuroblastoma cells.

## Materials and Methods

### Reagents

Tunicamycin (Sigma-Aldrich, MO, USA) was prepared in PBS (137 mM NaCl, 2.7 mM KCl, 4.3 mM Na_2_HPO_4_, 1.47 mM KH_2_PO_4_) as a 10 mM stock solution with 5% (v/v) DMSO at pH 7.4.

4-Phenyl butyric acid (PBA) and 5-Phenyl valeric acid (PVA) were purchased from Sigma (Sigma-Aldrich, MO, USA), and 10 mM solutions were prepared by titrating equimolecular amounts of PBA or PVA with sodium hydroxide to pH 7.4.

### Cell Culture

The SH-SY5Y cell line was obtained from ATCC (CRL-2266) [21] and cultured in 35 mm (for RNA isolation) or 60 mm (for protein isolation) plates (Becton Dickinson, NJ, USA). The cells were grown to 90% confluence at 37°C in an atmosphere of 5% CO_2_ and in Dulbecco's modified Eagle's medium (DMEM) supplemented with Glutamax (Gibco, Invitrogen, CA, USA), 100 units/ml penicillin/streptomycin, 1× MEM non-essential amino acids and 10% fetal bovine serum (FBS).

The HEK-293T cell line was obtained from ATCC (CRL-1573) [22] and the cells were grown in DMEM supplemented with 2 mM L-glutamine, 1 mM sodium pyruvate, 100 units/ml penicillin/streptomycin, 1× MEM non-essential amino acids and 5% (v/v) FBS. All the media and supplements were purchased from Invitrogen, (CA, USA).

### Cell death assay

To establish the optimal concentration of tunicamycin that produces a measurable amount of SH-SY5Y cell death, the cells were treated with different concentrations of this drug for 48 h and cell death was monitored by quantifying lactate dehydrogenase (LDH) release into the cell media with the Cytotoxicity Detection Kit (Roche Diagnostics IN, USA). The analysis was carried out according to the manufacturer's recommendations and the absorbance was measured at 450 nm with a microplate reader.

To assess the effects of PBA or PVA on survival, SH-SY5Y cells were exposed to these compounds for 24 h at the concentrations indicated and they were then treated with 500 nM tunicamycin for 48 h.

### Quantitative Real-Time PCR

Total RNA was extracted from SH-SY5Y or HEK-293T cells using a method based on that of Chomczynski and Sachi's [23] and the TRI reagent (Sigma-Aldrich, MO, USA). Briefly, cells were washed with PBS and lysed with 1 ml Trizol reagent, and after a 5 min incubation at room temperature, the lysate was mixed vigorously with 0.2 ml of chloroform. The sample was then centrifuged at 12,000 g for 15 min at 4°C, and the supernatant was recovered and placed in a fresh tube containing 0.5 mL isopropanol. After incubating for 10 min at room temperature, the RNA pellet was obtained by centrifugation at 12,000 g for 10 min at 4°C. The pellet was washed in 1 mL ethanol (75%) and once all the ethanol had been removed, it was dissolved in 30 µl diethyl pyrocarbonate (DEPC)-treated water.

2 µg of total RNA obtained was used as a template to synthesize cDNAs with the SuperScript® III First-Strand Synthesis System for RT-PCR (Invitrogen, Life technologies). Real-time quantitative PCR assays were then performed in triplicate on these cDNAs in the presence of the PCR Master Mix (Power SYBR®Green, Applied Biosystems, Warrington, UK) to detect the amplification products. Samples were analyzed simultaneously for ribosomal protein 36B4 mRNA as an internal control using an ABI Prism 7300 sequence detector (Applied Biosystems, Foster City, CA, USA), and the data were analyzed using Sequence Detection software v. 3.0. (Applied Biosystems). The primer sequences for quantitative PCR are indicated in Table 1.

**Table 1:** Primer sequences used for quantitative PCR.

**36B4 up**	AACATCTCCCCCTTCTCCTT
**36B4 down**	GAAGGCCTTGACCTTTTCAG
**GRP78 6**	ACCAACTGCTGAATCTTTGGAAT
**GRP78 5**	GAGCTGTGCAGAAACTCCGGCG
**XBP1 splicing**	CGGGTCTGCTGAGTCCGCAGCAG
**XBP1 total**	GCAGGTGCAGGCCCAGTTGTCAC
**XBP1**	CCCCACTGACAGAGAAAGGGAGG
**ERdJ4 187**	GAAAACTCCTGGAAGTGATGCCTTTGTCTA
**ERdJ4 186**	TCACAAATTAGCCATGAAGTACCACCCTGA
**ASNS 238**	TTGGGTCGCCAGAGAATCTCTTTGGG
**ASNS 237**	GTATATTCGGAAGAACACAGACAGCGTGG
**CHOP Fw**	GCTGGGAGCTGGAAGCCTGGTATG
**CHOP Rev**	TCCCTGGTCAGGCGCTCGATTTCC

### Protein extracts

Total protein homogenates were obtained by homogenizing the cells in ice-cold lysis buffer (200 mM NaCl, 5 mM EDTA, 100 mM HEPES pH 7.4, 10% glycerol, 2 mM Na_4_P_2_O_7_, 1 mM EGTA, 2 mM DTT, 0.5 mM phenylmethylsulfonyl fluoride and the CompleteTM Protease inhibitor cocktail [Roche Diagnostics, Mannheim, Germany]) that contained phosphatase inhibitors (1 mM Na_3_VO_4_, 200 mM NaF). The homogenates were disrupted by passing them through a 27G needle and they were then left on ice for 30 min before they were centrifuged at 11,000 g for 20 min at 4°C. The protein concentration was determined using the Bradford assay (Bradford protein assay, Bio-Rad, CA, USA) and aliquots were stored at −80°C.

### Immunoblotting

Protein samples were mixed with 6× Laemmli sample buffer resolved on 10% SDS-polyacrylamide gels and transferred to nitrocellulose membranes. The membranes were blocked with 5% milk in Tris-buffered saline (TBS –50 mM Tris, 150 mM NaCl pH 7.4) containing 0.05% Tween-20 and then probed overnight with primary rabbit polyclonal antisera against: rabbit polyclonal phospho eIF2α (1∶1000 Cell Signaling Technology, Beverly, MA), rabbit polyclonal anti-ATF4(1∶1000 Santa Cruz Biotechnology CA, USA), rabbit polyclonal anti caspase 3 (1∶300 Cell Signaling Technology, Beverly, MA) and mouse polyclonal anti-actin (1∶2000 Sigma-Aldrich, MO, USA). Following two washes in TBS/Tween20 and one in TBS alone, the immunolabeled proteins were detected with a HRP-conjugated anti-rabbit or anti-mouse antibody (1∶5,000 Santa Cruz Biotechnology), which was visualized by enhanced chemiluminiscence (ECL, GE Healthcare Bioscience, Buckinghamshire,UK) and autoradiography using HyperfilmTMECL (GE Healthcare Bioscience). Quantity OneTM software v.4.6.3 (Bio-Rad) was used for quantification.

### Data analysis and statistical procedures

The results are reported as the means ± SEM and they were analyzed with the SPSS package for Windows, version 15.0 (SPSS, Chicago, IL,USA). All measurements were taken in triplicate from three independent experiments. The Shapiro Wilks test was used to evaluate the fit of the data to a normal distribution and the Levene test to evaluate the homogeneity of variance. Significance was tested using one-way ANOVA followed by a Tukey's multiple range test.

## Results

**Figure 2:**
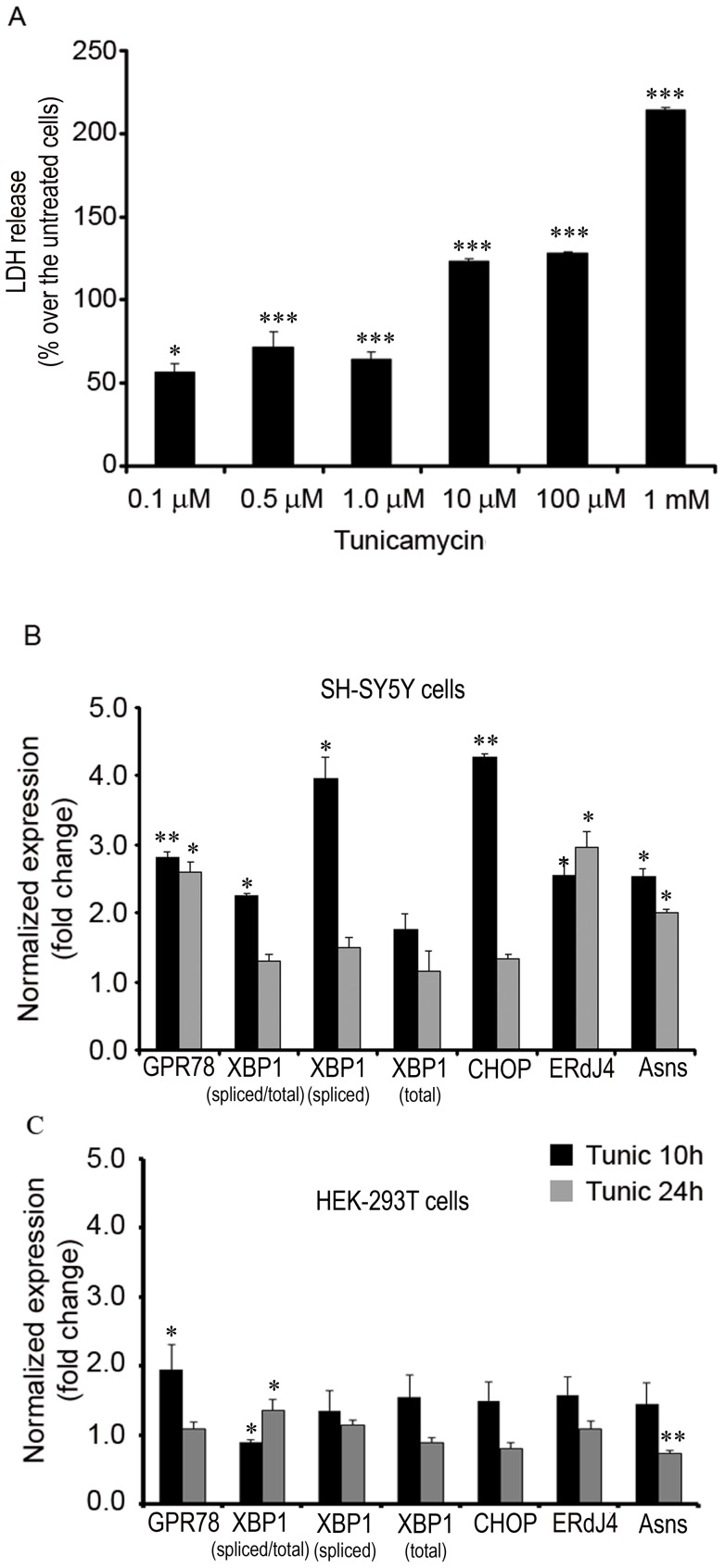
Tunicamycin-induced ER stress in SH-SY5Y cells. A) SH-SY5Y cells were treated for 48 h with the indicated concentrations of tunicamycin and cell viability was determined using a LDH release assay. Bars represent the percentage of LDH release over that obtained in untreated cells: *p<0.05; ***p<0.0001 *vs* untreated cells. B–C) The transcriptional activity of different sensors was used to monitor the induction of ER stress by tunicamycin in SHSY-5Y (B) and HEK-293T (C) cells. Bars represent the fold change (mean ± SEM) in gene expression normalized to the control untreated cells: *p<0.05; **p<0.005; *vs* control untreated cells.

The effect of the ER stress inducer, tunicamycin, on cell death measured by the LDH activity released to the culture medium was assayed in human neuroblastoma SH-SY5H cells. We observed a dose-dependent increase in cell death that reached a plateau at concentrations of 0.5 and 1 µM tunicamycin (Fig. 2A). Higher concentrations of tunicamycin (10 or 100 µM) caused massive cell death in the cultures. Accordingly, the tunicamycin concentration selected to measure the activation of components of the UPR and the neuroprotection afforded by the phenyl acyl acid compounds was 500 nM. When the expression of selected UPR mediators was measured by RT-PCR, exposure to tunicamycin increased the mRNA expression of all of them: spliced XBP-1 and spliced XBP-1/total ratio, CHOP, endoplasmic reticulum-localized DnaJ homologue 4 (ERdJ4) and asparagine synthetase (ASNS). Moreover, the cytotoxic effect of tunicamycin was rapid as it was stronger at 10 h than at 24 h (Fig. 2B). A similar experiment was performed with HEK-293T cells, in which UPR activation has been documented previously, yet the enhanced expression of these UPR substrates induced by tunicamycin (500 nM) was not as pronounced as in neuroblastoma cells (Fig. 2C). These results suggest that neuroblastoma cells are more sensitive to tunicamycin than other cells for which concentrations up to 0.125 mM have been used to measure stress-related effects [24].

**Figure 3:**
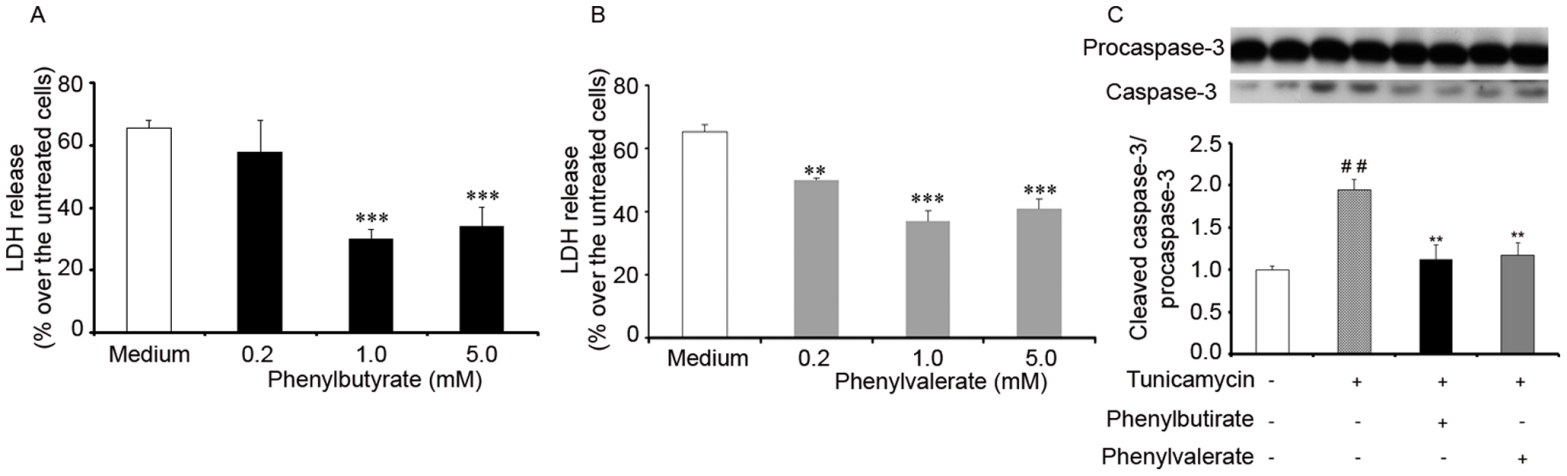
PBA and PVA protect SH-SY5Y cells against tunicamycin induced ER stress. SH-SY5Y cells were exposed to tunicamycin (500 nm) for 24 h in the absence (medium) or in the presence of PBA (panel A) or PVA (panel B). Cell viability was determined using a LDH release assay and the results are expressed as the means ± SEM. Bars represent the percentage of LDH release over that obtained in untreated cells. **p<0.005; ***p<0.0001 *vs* tunicamycin treated cells (Medium). Panel C. Immunoblot of procaspase 3 and cleaved caspase 3. A representative image is shown and the bar diagram represents the ratio of cleaved versus total protein (mean ± SEM) in the different conditions and normalizad to the ratio in absence of tunicamycin. ^##^p<0.005 *vs* untreated cells; **p<0.005 *vs* tunicamycin treated cells.

**Figure 4:**
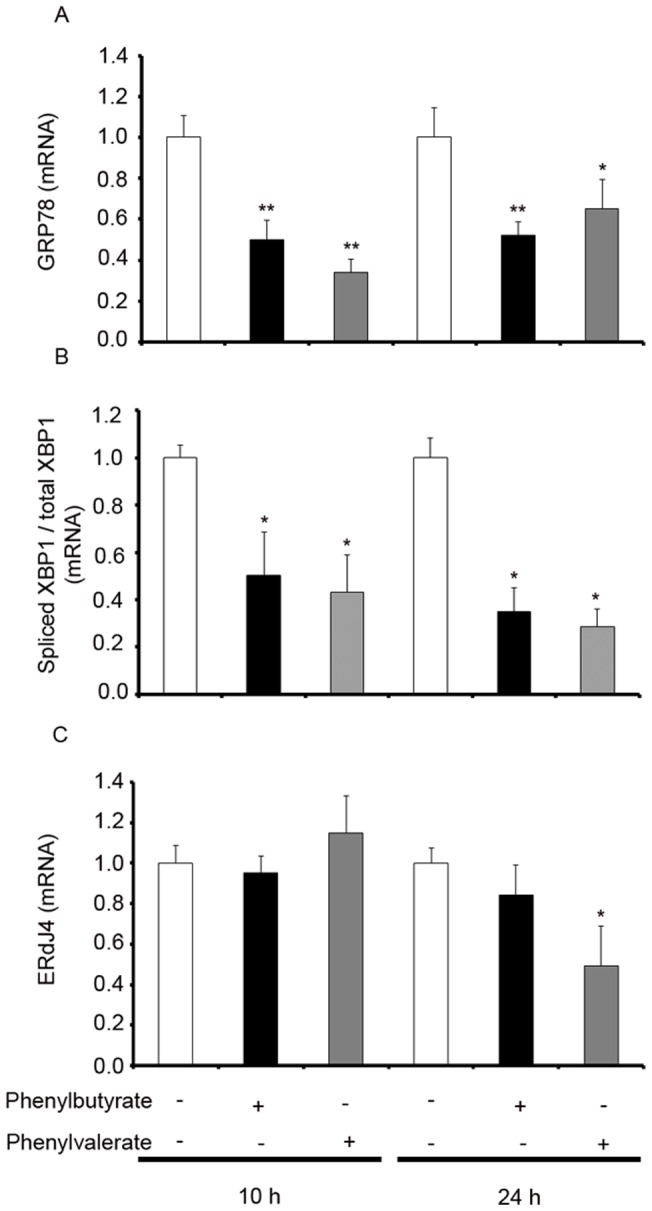
PBA and PVA neutralize the ER stress sensors induced by tunicamycin. SH-SY5Y cells were treated as described in the Materials and Methods and the expression of GPR78 (A), spliced XBP1 (B) and ERdJ4 (C) was determined by RT-PCR. The bars represent the expression (mean ± SEM) normalized to that of the corresponding 36b4 internal control: *p<0.05; **p<0.005 *vs* tunicamycin treated cells.

Significant protection against the cell death provoked by a submaximal 10 h and 24 h exposure to tunicamycin was offered by PBA or PVA (see Methods). LDH measurements indicated that both PBA and PVA offered dose-dependent protection against neuroblastoma cell death that reached a plateau at low mM levels in both cases (Fig. 3A–B). The levels of proapoptotic active caspase 3 were also determned and the results indicated both that tunicamycin increased the levels of the cleaved form of the enzyme and that PBA or PVA reverted them to the values obtained with media alone (Fig. 3C). The treatment of cells with PBA or PVA (1 mM) significantly impaired the tunicamycin-induced upregulation of the ER stress marker, BiP, also known as the glucose-regulated protein 78 (GPR78: Fig. 4A). As indicated earlier, stress-mediated IRE-1 activation (such as that produced by tunicamycin) leads to the specific splicing of the mRNA encoding XBP-1 [25], a modification that was significantly diminished by both PBA and PVA (Fig. 4B). Indeed, after 10 h in the presence of tunicamycin, PBA or PVA-treated cells displayed a two-fold decrease in the levels of spliced XBP1 mRNA, a reduction that was even more pronounced after 24 h of treatment (Fig. 4B). The protein translated from the spliced XBP-1 mRNA, Xbp1s, controls the expression of the ER co-chaperone ERdj4, which is upregulated by ER stress. Indeed, this protein has been implicated in ER-associated degradation (ERAD) of multiple unfolded secretory proteins. When the effect of PBA and PVA on ErdJ4 mRNA expression was analyzed, the increase in expression provoked by a 10 h treatment with tunicamycin (Fig. 2B) was not modified by either compound. However, while PVA provoked a significant decrease in expression at 24 h, the effect of PBA was not significant (Fig. 4C).

**Figure 5:**
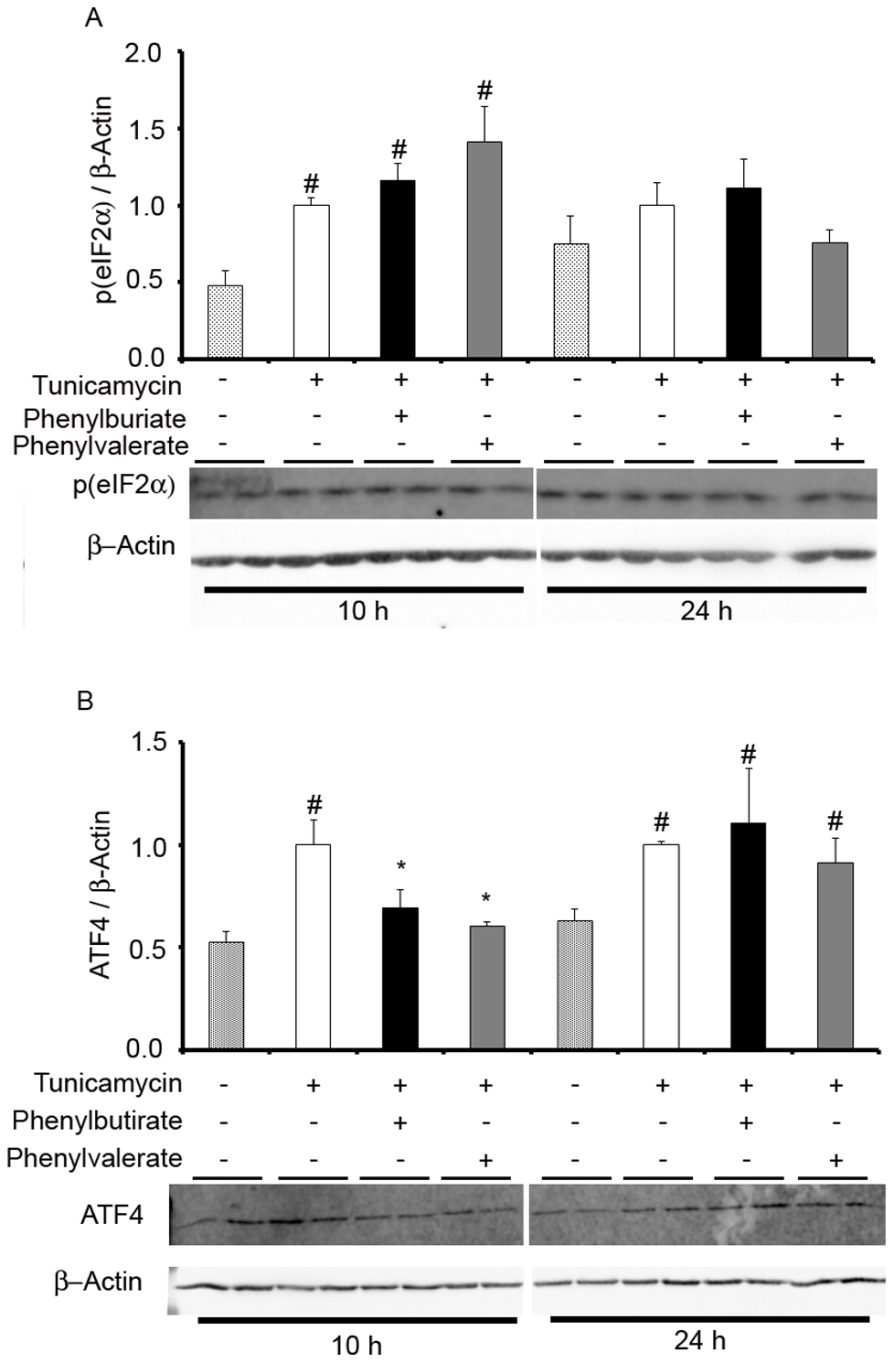
Western blots of SHSY-5Y cells treated with PBA or PVA probed with anti-phospho eIF2α (A) and anti-ATF4 (B). The bars represent the ratio of eIF2α or anti-ATF4 versus β-actin expression and referred to the ratio in tunicamycin-treated cells (mean ± SEM). *p<0.05, *vs* tunicamycin treated cells.^#^p<0.05 *vs* untreated cells.

**Figure 6:**
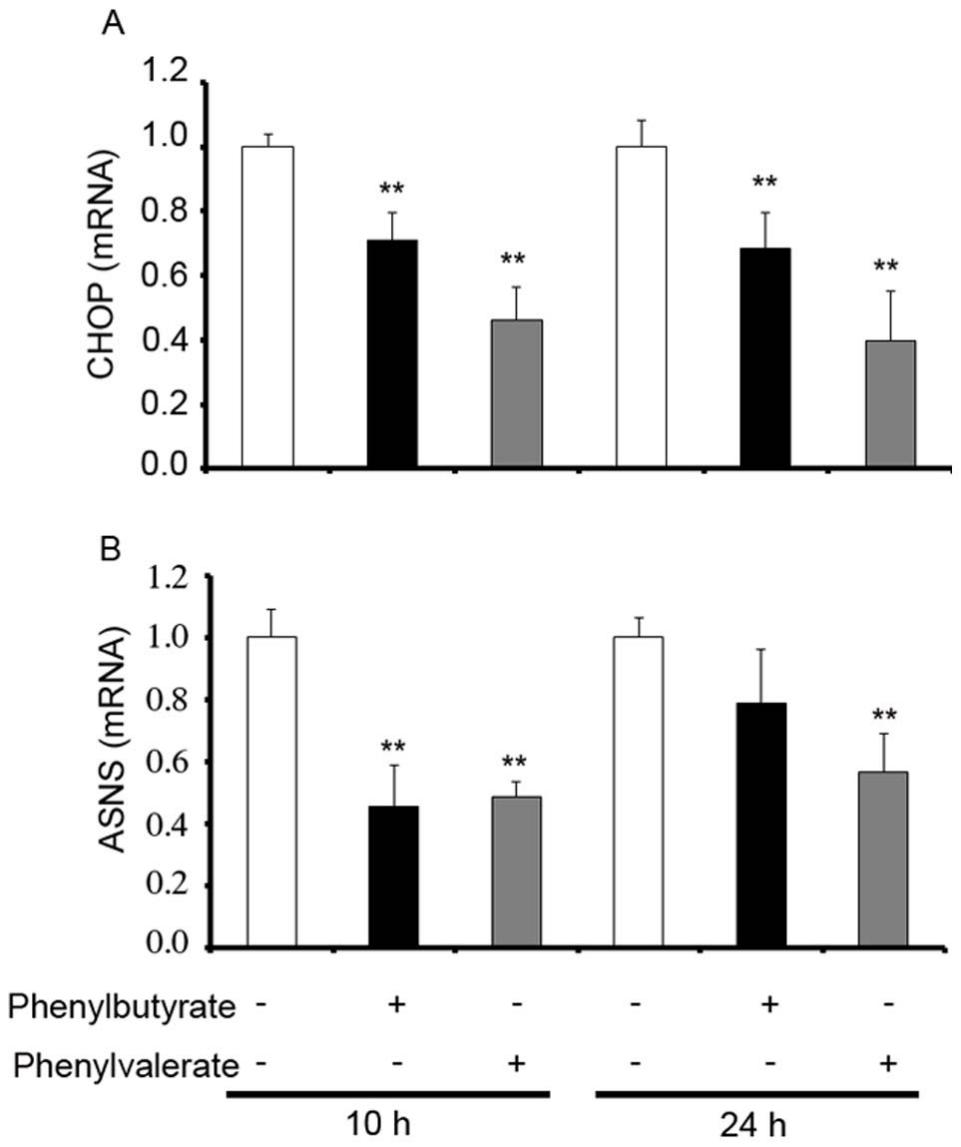
Induction of CHOP and ASNS expression in SHSY-5Y tunicamycin ER stressed cells is reversed by PBA or PVA treatment. Cells were treated for 24 h with tunicamycin and with PBA or PVA for the times indicated. The expression of CHOP and ASNS was determined by RT-PCR and normalized to that of the corresponding 36b4 internal control. Bars represent the mean ± SEM of the relative change with respect to tunicamycin treated cells: **p<0.005 *vs* tunicamycin treated cells.

PERK signaling is also activated under conditions of ER stress and it augments the expression of the activating transcription factor 4 (ATF4), which is in turn dependent on the phosphorylation status of the translation initiation factor eIF2α. Enhanced phosphorylation of eIF2α triggered by 10 h tunicamycin treatment was not further increased by either PBA or PVA (Fig. 5A). However, expression of eIF2α was similar to that of control after 24 h of treatment with tunicamycin (with or without PBA or PVA). In contrast, PBA or PVA were able to revert the significant increase of ATF4 expression by 10 h-treatment with tunicamycin (Fig. 5B). This PBA or PVA effect was not observed at the later time (Fig. 5B). Although PERK-dependent eIF2α phosphorylation is known to shutdown translation, mRNAs containing upstream open reading frames (uORFs) can bypass this block, as occurs with ATF4, which in turn activates ASNS transcription. A similar effect occurs with the pro-apoptotic protein CHOP, which is also transcriptionally activated by ATF4. Significantly, PBA and PVA decreased the expression of CHOP at both time points when measured by RT-PCR (Fig. 6A), and ASNS expression was also significantly diminished, except when cells were exposed to tunicamycin for 24 h following treatment with PBA (Fig. 6B). Together, these results indicate that PBA and PVA do not affect PERK signaling at the level of eIF2α phosphorylation but rather, after 10 h of tunicamycin-induced stress they significantly decrease ATF4 expression, leading to the downregulation of ASNS and CHOP expression. Moreover, PVA drives this effect more markedly than PBA.

## Discussion

The results presented here suggest that modulation of the ER stress responses may offer protection against neural cell death, such as that provoked by both PBA and PVA. As a chemical chaperone, PBA is a well-known modulator of proteostasis, and *in vitro*, PVA has also been shown to act as a chemical chaperone [14]. PBA and PVA may in part modulate the tunicamycin-induced stress response by diminishing the amount of unfolded proteins due to their intrinsic chemical chaperone activity. However, the modulation of stress-related gene expression, which was similar for PBA and PVA, may also be caused by other overlapping mechanisms. It was shown that in an early-onset model of Alzheimer's disease, the severity of the amyloid pathology is related to the pronounced dysregulation of histone acetylation in the forebrain, and that recovery of memory function was correlated with elevated hippocampal histone acetylation and enhanced expression of genes implicated in associative learning [26]. When either sodium butyrate or PBA are used, the cognition-enhancing effects and the regulation of histone deacetylation may be related [27]–[32]. Whereas sodium butyrate and PBA positively influence the transcriptional regulation of cognition-related genes, as reported here, PBA may also negatively regulate gene transcription [33]. In fact, PBA and PVA appear to be more closely associated with a decrease in the expression of factors upregulated in response to stress. Under the specific cell culture conditions used here, SH-SY5H cells invoke a complex transcriptional response to tunicamycin that involves the three UPR signaling branches. It is particularly notable that 10 h after such stress is triggered a large amount of mRNA for spliced XBP-1 and for CHOP accumulates. Interestingly, both PBA and PVA negatively modulate stress-related transcription, and while this response was qualitatively and quantitatively similar for both aromatic acyl acids, the downregulation provoked by PVA on ERdJ4 and ASNS transcription was more sustained than that of PBA. Nevertheless, this difference in UPR modulation dynamics does not seem to be important for neuroprotection, as both offered similar levels of neuroprotection. Together these results show a correlation between the neuroprotection afforded at 24 h with the negative regulation of stress-gene transcription that peaked at 10 h.

Under similar experimental conditions the stress response elicited in neuroblastoma cells was stronger than in HEK-293T cells, which may explain why studies using non-neuronal cells are performed with higher concentrations of tunicamycin. In highly tunicamycin-sensitive SH-SY5H cells, the decrease in the mRNA transcripts from stress genes was correlated with stronger neuroprotection, suggesting that in this neural cell model the negative modulation of ER stress/ER stress signaling 10 h after stress is triggered benefits survival. In fact, at this time both PBA and PVA significantly reduced the expression of GPR78, spliced Xbp-1, ERdJ4, CHOP and ASNS mRNAs. Furthermore, while the decrease in ERdJ4 expression would expected as a consequence of downregulating XBP-1 splicing, the weaker expression of CHOP and ASNS was correlated with the loss of the ATF4 protein. Interestingly, the downregulation of PERK signaling was not correlated with decreased phosphorylation of the eIF2α translation initiation factor by PERK. This may be due to a delay between mRNA downregulation and that of protein phosphorylation. In fact, an increase in eIF2α phosphorylation seems to be required to produce the switch in the translation machinery that promotes the synthesis of mRNA coding for PERK-specific factors (ATF4, CHOP and ASNS) [34]. Alternatively, the activation of CHOP or ASNS in neurons may not be entirely dependent on the PERK pathway, although this would be in contrast with findings in human hepatoma cells [35], in which transcriptional induction of the ASNS gene during the unfolded protein response requires the PERK but not the ATF6 and IRE1/XBP1 arms of the stress pathway.

Events associated with ER-stress have been linked to the pathogenesis of Alzheimer's disease, which is characterized by the accumulation of amyloid-derived products. In fact, rescue the ER-stress-induced suppression of amyloid precursor protein has been shown to be one of the beneficial actions of PBA, thereby preventing apoptosis in neuroblastoma NAG cells [36]. Interestingly, the intracellular domain of the amyloid precursor protein (AICD) enhances the sensitivity of human SHEP neuroblastoma cells to apoptosis induced by tunicamycin [37]. Therefore, it seems that it may be beneficial to address ER-stress in pathological conditions in which proteins accumulate in the CNS (e.g., Alzheimer's or Huntington's disease, tauopathies or synucleinopathies). The present report shows that both PBA and PVA dampen the activation of genes involved in the ER stress response caused by inhibiting N-linked protein glycosylation. Tuning down ER stress or ER stress-derived transcription soon after ER stress occurs may be critical to mitigate the toxic challenge. The contribution of PBA or PVA to neuroprotection via this effect merits attention.
